# The Learning Design & Course Creation Workshop: Impact of a Professional Development Model for Training Designers and Creators of Online and Distance Learning

**DOI:** 10.1007/s10758-022-09639-1

**Published:** 2023-01-11

**Authors:** Tom Olney, Bart Rienties, Daphne Chang, Duncan Banks

**Affiliations:** 1 Faculty of Science, Technology, Engineering and Mathematics, the Open University, MK76AA Milton Keynes, UK; 2 Institute of Educational Technology (IET), the Open University, MK76AA Milton Keynes, UK

**Keywords:** Professional Development, Learning Design, Online and Distance Learning

## Abstract

Higher education institutions are increasingly moving from traditional education approaches to incorporate online and distance learning (ODL) models, and this represents a substantial educational challenge for many educators. One way to support this challenge is by providing appropriate professional development (PD) for the design of ODL. Based on models from the Open University, UK this paper contends that PD for the design of ODL should align learning design frameworks with constructivist and student-focused pedagogies to manage the change to the professional teaching identities of participants that designing ODL represents. Robust professional identities are important for mitigating anxiety, embedding lasting change and leveraging the benefits of ODL for institutions and students. In this study, the rationale, model, and strategies of the Learning Design and Course Creation Workshop, based on current literature and participant feedback gathered immediately after instances of the PD was completed in China, are described. Evidence and examples of impact gathered from a second instrument, gathered after implementation, is also provided, and discussed in this context. The findings and conclusions will be of interest to those tasked with providing PD to support educators faced with rapid educational change, particularly as a response to the global demand for the design of ODL.

## Introduction

Distance education has been defined as education in which an institutionally based interaction between educator, learner and content/resource exists, despite the educator and learner being physically separated. The medium of this interaction has evolved through five generations, which have been identified as (1) postal correspondence, (2) broadcast radio and TV, (3) open universities, (4) teleconferencing, and (5) the internet (Moore & Kearsley, 2011). Distance education that takes place in the fifth-generation, via the internet, can be referred to as ODL (Martin et al., 2022). Distance education has been well-established in such large and culturally diverse higher education institutions (HEI) as the Open University UK (UKOU), the Open University of China (OUC) and University of South Africa (UNISA). These HEI, amongst many others, have been leading on incorporating ODL into their fifth-generation offerings for students in recent years.

ODL is attractive to HEI because it is seen as cost effective, able to reach learners who might otherwise be unable to access a campus-based course and offers education in places and times where it might not previously have been possible (Means et al., 2013). Regarding student outcomes, recent studies have demonstrated that ODL can deliver better results in areas such as cognitive learning and quantitative reasoning (Martin et al., 2022).

During the COVID-19 pandemic, previously co-located educators and learners in traditional HEI were forced apart, to slow transmission rates of the virus, and these institutions, educators and learners had to quickly adapt, or ‘pivot’, to ODL approaches (Weller, 2020). As a result, international guidance for policy makers on measures to support educators with the impact of COVID-19 was published. Targeted PD and a review of ‘development frameworks to update provisions for distance education’ was required in order ‘to strengthen the resilience of education systems’ (UNESCO & ILO, 2020: 4). Developing systems and resources – via PD, for example – ‘…that can be leveraged in times of shock when core delivery models are disrupted’ is vital for future sustainability (Portillo and Lopez de la Serna, 2020: 3). Hodges et al., (2020) point out that the student experience is meaningfully improved in well-designed ODL courses rather than those hastily offered online in response to a crisis or disaster.

An increasing percentage of educators and executive leaders in HEI believe ODL will be a fundamental component of their future teaching and learning offerings (JISC, 2020), but research also suggests that substantial gaps exist between the perceived skills and competencies of educators to design and implement ODL approaches, and the PD available to them (Roberts, 2018; JISC, 2020, (Olney et al, 2021a). Whilst preparation for moving to ODL approaches is considered urgent (Heap et al., 2021) challenges are prevalent in areas such as mitigating high levels of educator anxiety (JISC, 2020), supporting changing professional teaching identities (Philipsen et al., 2019) and, improving perceptions of quality (Olney et al, 2021a; Li & Chen, 2019). This paper outlines a model for delivering appropriate PD for the design of ODL that addresses challenges such as these.

The Learning Design and Course Creation (LDCC) Workshop is a model of PD that synthesises ODL educational principles and examples of practice currently in use at the UKOU. This paper provides a theoretical rationale for the model, based on current literature and participant feedback gathered immediately after completion of the PD, which aligns learning design (LD) frameworks with constructivist and student-focused pedagogies to support the changing professional teaching identities of participants. It also provides some evidence and examples of impact collected one to six years later, after opportunities for implementation have occurred. The research question guiding this study is:

*Can PD, which aligns LD frameworks with constructivist and student-focused pedagogies, support the changing professional identities of teachers when they are tasked with designing ODL?*

## Literature Review

A recent comprehensive literature review by Philipsen et al., (2019) of 15 articles written between 2005 and 2014 on PD programs that target approaches to distance education highlighted a scarcity of relevant research. It found that whilst there is a great deal of research on delivering PD online, PD for the design of blended and online distance education was not well represented in the literature (Philipsen et al., 2019). Despite this, the authors developed a PD framework that consisted of six components based on the synthesised findings of the 15 studies. The framework identified these components as: (i) developing supportive environments, (ii) acknowledging existing contexts, (iii) determining clear and relevant goals, (iv) adopting strategies to encourage reflection, active learning and peer support, (v) establishing ongoing evaluation, and, in particular, emphasised the importance of, (vi) addressing changes to the professional identity and educational beliefs of teachers.

Common features of studies that focus on PD for the design of ODL place emphasis on the establishment of collaborative teams as being beneficial for building supportive environments and developing confidence in the participants (Koehler et al., 2004; Nihuka & Voogt, 2012; Peeraer & van Petegem, 2012; Rienties et al, 2013; Young & Perovic, 2016). Similarly, another commonly referenced feature seen as being beneficial to the design of ODL is the use of concrete templates and procedures (Nihuka & Voogt, 2012; Young & Perovic, 2016, Co-Designs, 2020; Olney & Piashkun, 2021b).

However, in a quantitative meta-analysis of research on the effectiveness of PD for design for academics Ilie et al., (2020) found that neither the types nor focus of activities engaged with during a PD had a large effect on the quality of the design practice of the participants. Rather, their analysis suggested that non-pedagogical elements such as the duration of the PD or mandatory enrolment were the most influential factors. The authors concluded that future work should contribute by describing similar PD in detail and ‘adding more information regarding the core characteristics’ (Ilie et al., 2020: 15).

In a 2018 study of distance educators, staff at leading ODL HEI, UNISA, perceived themselves as having low levels of competency in the roles of technology expert and instructional designer, when compared with other roles such as knowledge expert, and self-identified a need for increased levels of future PD to support these roles (Roberts, 2018).

Similarly, a recent review (Uerz et al., 2018: 18) found a consensus exists in the ODL PD literature that ‘technological proficiency in itself is no guarantee of pedagogical proficiency in educational technology’ and that teachers must first learn to use educational technologies within their own contexts if they are ever to implement them successfully. In a study of 99 teachers in Spain, Guillen-Gamez et al. (2021) recommended that PD should not treat educational technologies as independent content but that they should be at the service of learning and teaching strategies. Several studies that explore developing PD for ODL also point to the importance of reviewing and applying what has been learnt into the specific situation and context of practice within the scope of the PD (Mittelmeier et al., 2018; Portillo & Lopez de la Serna, 2020; Olney & Piashkun, 2021b).

Uerz et al., (2018) note how several studies highlight the importance of the relationship between beliefs about teaching and learning and changes to pedagogical practice and how the integration of educational technologies goes along with opportunities for teachers to change from teacher-focused to student-focused situations. However, it also found that the current literature was ‘ambiguous’ about the nature of the relationship between the beliefs of teacher educators and the use of technology in teaching. In order to establish how one influences the other the authors concluded, ‘…further research is needed’. (Uerz et al., 2018: 21).

## Methods

### Background and Settings

#### Learning Design Frameworks

Whilst a widely recognized and accepted definition for LD remains elusive, some useful concepts and frameworks exist that can be utilized to help explain the key features. Orientating LD can be problematic because the term has evolved to describe it occupying at least three distinct roles (Dalziel et al., 2016) which in turn affect the way it is perceived by different stakeholders (Godsk, 2018). These roles include considering LD as:

(i) A product, that is: ‘a’ learning design – a plan or recorded sequence of teaching and learning activities​.
(ii) A process, that is: one or more events or stages that are attended or completed to assist in the development of a piece of teaching and learning. ​.
(iii) A practice, that is: the action of applying LD concepts to the creation and implementation of a piece of teaching and learning. ​.

Whilst all three orientations need to be recognised, the focus of this paper is on considering ways in which to introduce and facilitate LD into the practice of educators who are tasked with designing and creating ODL, since ‘…building design capacity in teachers offers opportunities for large-scale, sustainable change’ (Bennett et al., 2018; 1015). Conceptually, LD provides a mechanism for this, but makes no claims as to the method in which this should be done. LD frameworks are characterised by the three underpinning concepts of representation, sharing and guidance and are perhaps best understood as being able to ‘…describe a broad range of teaching and learning activities…’, or ‘…aspiring towards being pedagogically neutral’ rather than being a traditional pedagogical theory (Dalziel et al., 2016; 9).

#### Constructivism and Student-focused Learning in Professional Development Environments

In constructivist approaches learning is acquired by creating meaning from experience and these experiences can be facilitated by involvement in authentic tasks anchored in meaningful, real-life contexts. Therefore, a constructivist PD environment for the design of ODL might be one which emphasises: the importance of the context in which the learning is taking place and will be applied; the situating of tasks as working towards solving practical, real-world problems; the learner choosing how to apply what is being learnt; presentation of information and genuine examples in multiple ways; social negotiation as a way to establish meaning (Ertmer & Newby, 2013).

Since constructivist environments position the teacher as a facilitator or ‘guide’ (Ertmer & Newby, 2013) the responsibility, accountability, and autonomy of the student in their own learning process becomes more important and the relationship between the teacher and the learner becomes one of interdependence rather than dependence, forming the characteristics of a student-focused approach to learning (Lea et al., 2003). In a PD for the design of ODL it therefore follows that consideration of the learners needs, capabilities and environments must be recognised and their acquisition of skills, rather than knowledge, becomes paramount. Research on 228 Turkish teachers by Senturk & Bas (2021) found a positive relationship between teachers who held constructivist beliefs towards teaching and learning and their attitudes towards the benefits of educational change as represented, for example, by the introduction of ODL. This finding resulted in a call for all Turkish educational PD to be situated in both physical and pedagogical constructivist learning environments (Senturk & Bas, 2021).

#### Professional Identity of Teachers in Professional Development Environments

Some key concepts that explain how the professional identity of teachers are formed and mature are anchored in their perceptions and beliefs about education, pedagogy and the fundamental role of the teacher. This study set out to relate the concepts of *task perception* and *subjective educational theory* which, when taken together, can operate ‘as a lens through which teachers look at their job, give meaning to it and act in it’ (Kelchtermans, 2009: 260) to the design of ODL. A study of eleven academics over six years from Finland utilised these concepts to reveal that PD that sought to build reflective practice and peer interaction assisted most in the development of a robust teacher identity (Nevgi & Lofstrom, 2015).

The concept of *task perception* deals with the normative question of ‘what must I do to be a proper teacher?’ *Task perception* reflects the reality that teachers are constantly challenged to make value judgments about what is included in their professional programme of tasks, duties, and activities and their ‘…deeply held beliefs about what constitutes good education’ (Kelchtermans, 2009: 262). If calls for educational change contradict the *task perception* of teachers, and workable alternatives are not provided, they can negatively affect self-esteem, job satisfaction and may even result in burnout.

A teacher’s related *subjective educational theory* is described as ‘the personal system of knowledge and beliefs about education that teachers use when performing their job…and the basis on which teachers ground their decisions for actions’ (Kelchtermans, 2009: 264). This concept builds on the ‘what must I do to be a proper teacher?’ question posited by *task perception*, to further question: ‘how can I become a proper teacher?’ For knowledge or beliefs to take root in the *subjective educational theory* of teachers they must experience that ‘it works for them’ or is ‘true for their practice’ and can assist them in making judgements about acting in certain circumstances. In other words, it is influenced by the kinds of practical, real-world application encouraged by constructivism.

Therefore, the working hypothesis of this study was that PD for the design of ODL that aligns LD frameworks with strategies that model student-focused and constructivist pedagogies in their own design would maximise the likelihood of participants adopting these pedagogies into their own design for ODL practices where they have the agency to do so. Further, this model of PD for the design of ODL would support the *task perception* and *subjective educational theory* of the participants, providing them with the capacity and capabilities to successfully manage the educational change designing ODL represents.

#### Learning Design at the UKOU & the LDCC Workshop

The particular interpretation of LD that is currently in practice at the UKOU, and reflected in the LDCC Workshop, has its foundation in the findings from the OU Learning Design Initiative (OULDI) which ran from 2007 to 2012. The UKOU and 13 other higher education institutions participated in the *Institutional Approaches to Curriculum Design and Delivery* programme which was co-funded by the not-for-profit Joint Information Systems Committee (JISC) and the European Union (EU) (Conole & Wills, 2013). Wide ranging interviews with staff at these institutions revealed a multitude of design practices. As a consequence of the OULDI, since 2012 LD practitioners at the UKOU have sought to embed constructivist approaches that are student- focused and based around the three principles of:

i. encouraging design conversations and collaboration in design.
ii. using tools, instruments and activities to describe and share designs.
iii. developing LA approaches to support and guide decision-making.

In the daily life of the UKOU, LD workshops provide a mechanism for bringing together multi-disciplinary staff in teams to design new curriculum. Outputs from these workshops are then recognised as key components in an internal quality assurance process (Galley, 2015). The LDCC Workshop has been developed to incorporate student-focused and constructivist pedagogies into strategies that maximise the likelihood of participants adopting these same pedagogies into their own design for ODL practice and supporting changes to their *task perception* and *subjective educational theory*. The pedagogy of the LDCC Workshop provides a structured way to present design for ODL educational principles, tools, activities and examples of practice currently in use at the UKOU which, for simplicity, will be now referred to collectively as LDCC approaches.

#### ODL in China

Rapidly growing student numbers and an increasing demand for quality teaching in the ODL sector of Chinese Higher Education (HE) is driving rapid educational change (Qi & Li, 2018; Li & Chen, 2019; Zhang & Li 2019). To manage this change and meet Chinese Ministry of Education (MOE) directives a need for learning design, constructivist, and student-focused models of PD for the effective design of ODL has been identified (Guan & Meng, 2007; Zhu & Liu, 2020; Olney et al, 2021a). Whilst examples of collaborative models for PD in the Chinese primary sector have been developed, and the impact of them on the knowledge construction of Chinese educators has taken place (Zhang S et al., 2017, Zhang N et al 2020), the effectiveness and impact of importing constructivism and student-focused educational approaches into Chinese HE remains contentious and unproven. Several studies argue there is nothing culturally inherent that prevents Chinese adult learners from successfully adopting such approaches given appropriate time, support and justifications for the value of such approaches (Walker et al., 1996; Kember, 2000; Kennedy, 2002) whilst others highlight the fundamental challenges to the role of the teacher (i.e. *task perception* and *subjective educational theory)* in areas such as content mastery, teaching approach, and assessment (Guan & Meng, 2007; Tan, 2017).

### Participants, Instruments and Analysis

By Dec 2021 around 750 Chinese staff, from at least eight different institutions, had participated in 29 instances of the LDCC Workshop. Feedback was gathered from this group of participants using two different instruments (A & B) and at two different times. Names, ages, gender, or other demographic data was not collected since the participants were invited to the workshop by their employer and we wanted to gather honest, unbiased opinions from the largest number of participants possible. Due to this we were unable to draw direct links for individuals between the two instrument results. Responding to the instruments was encouraged but voluntary, which resulted in an incomplete data set and some self-reporting bias. However, the resulting sample sizes are still large enough for meaningful analysis. These limitations in the sample are acknowledged. All participants were assured of anonymity and the purpose of the feedback was explained to them.

#### Instrument A

Instrument A was a written self-reporting survey completed at the end of the LDCC Workshop designed to establish the immediate challenges facing the participants and contained two questions:

1. ‘How easy/difficult do you think it would be to implement the LDCC approaches into your branch or institution?’, with four options.
2. ‘In your opinion, what is the most important thing that would need to change, in order to make implementation easier?’, as an open-ended question.

Usable data (both questions answered) was collected from 254 participants from 5 different Chinese HEIs, who attended 11 LDCC Workshops that took place between Nov 2017 and July 2020. Data was translated from Mandarin into English by a qualified translator hired for the purpose. Where participants had identified more than one ‘important thing’ in response to question 2 these were separated. A previously co-developed thematic coding framework (Table 1) (Olney et al, 2021a) was employed to analyse the comments in NVivo 12 employing frequent checks for consistency and reliability.

**Table 1:** Thematic coding framework

Code	Description of theme	Qualification	Key terms
1	The ways teams are established and operated (teamwork)	*…for design purposes*	Team, teamwork
2	Technical systems (platform/website/IT)	*…technical systems or people that support ODL*	VLE, technical, platform, website, IT
3	Student-centred specified approaches to pedagogy or design	*…in relation to ODL teaching and learning and activity types classification framework*	Student-focused, -centred, -perspective, -first, -profile, -situation, activity types
4	Bureaucratic systems (organizational/institutional/national)	*…bureaucratic systems that support ODL*	Management, investment, structure, policy, leadership, administration, financial, hiring
5	Academic workload (more time or training)	*…for teachers to spend on design*	Workload, time, busy, training, expertise
6	Use of data and learning analytics	*…to analyse both design and learning*	Data, analytics, evaluation, assess, assessment, feedback
7	Assessment task design	*…design of tasks for student learning*	Assessment, task
8	Non-specified approaches to pedagogy or design	*…in relation to ODL teaching and learning*	Ideology, pedagogy, philosophy, concept, notion, quality, knowledge
9	Ability of students to learn	*…learning in ODL*	Ability
10	Designing learning outcomes	*…outcome focused learning and teaching*	Learning outcomes, objectives, goals
11	Engagement and motivation of students	*…factors external to teaching in ODL*	Engagement, motivation
x	Non-applicable	*…workshop specific or unclear*	

#### Instrument B

Instrument B was a self-reporting online survey designed to measure the impact of the LDCC Workshop on the design for ODL practice of the participants consisting of five Likert-scale questions and an open response question (see Tables 2, 3, 4 and 5). It was sent in October 2020 to 524 participants who had attended one of 21 LDCC workshops between Jan 2014 and July 2020. 334 participants received the survey link directly from the authors via email, or Chinese social media app WeChat. 190 received the survey from their own institution. 134 participants completed the survey which represented a response rate of around 25%.

Data was translated from Mandarin into English by a qualified translator hired for the purpose, downloaded from MS Forms, and manipulated in MS Excel. 61 participants responded with qualitative comments which were separated and coded in the same way as with instrument A

**Table 2:** ‘Have you implemented any of the LDCC approaches into your own practice?’

	Question response	% of responses
1	No, I do not think that any of the LDCC approaches are useful for my own practice	0%
2	No, I have not had time to implement any of the LDCC approaches into my own practice	4%
3	No, I have not had the opportunity to implement the LDCC approaches into my own practice	27%
4	Yes, I am currently working on implementing some of the LDCC approaches into my own practice	35%
5	Yes, I have already implemented some of the LDCC approaches into my own practice and am waiting to see how my students are going to react to the updated design	20%
6	Yes, I have already implemented some of the LDCC approaches into my own practice and have received positive feedback	14%

**Table 3:** If you selected any of the ‘yes’ options above please briefly describe which LDCC approaches you have implemented and, if applicable, any positive results

Code	Count
1. Teams and teamwork	3
2. Technical systems	14
3. Student-centred pedagogy	50
4. Bureaucratic systems	1
5. Time and expertise	1
6. Student data & learning analytics	13
7. Assessment design	2
8. Non-specific pedagogy	6
9. Ability of students to learn	0
10. Designing learning outcomes	10
11. Engagement of students	10
12. Non-applicable	9
Total	**119**

**Table 4:** After attending the LDCC Workshop I believe that the LDCC approaches can support practitioners to more effectively - check all that apply

Options	No. of responses
Describe and share teaching and learning	62
Design student-centred learning	119
Evaluate the effectiveness of learning	71
Collaborate with colleagues	55
Improve student retention	31
Increase student achievement	28
None of the above	1
Other	0

**Table 5:** To what extent has the LDCC workshop you attended changed the way you think about designing ODL?

Response	No. of participants
None	0
Only a little	5
Moderately	38
Quite a lot	51
A lot	40
Total	**134**

## Results and Discussion

### Instrument A: Immediate Challenges

**Fig. 1:**
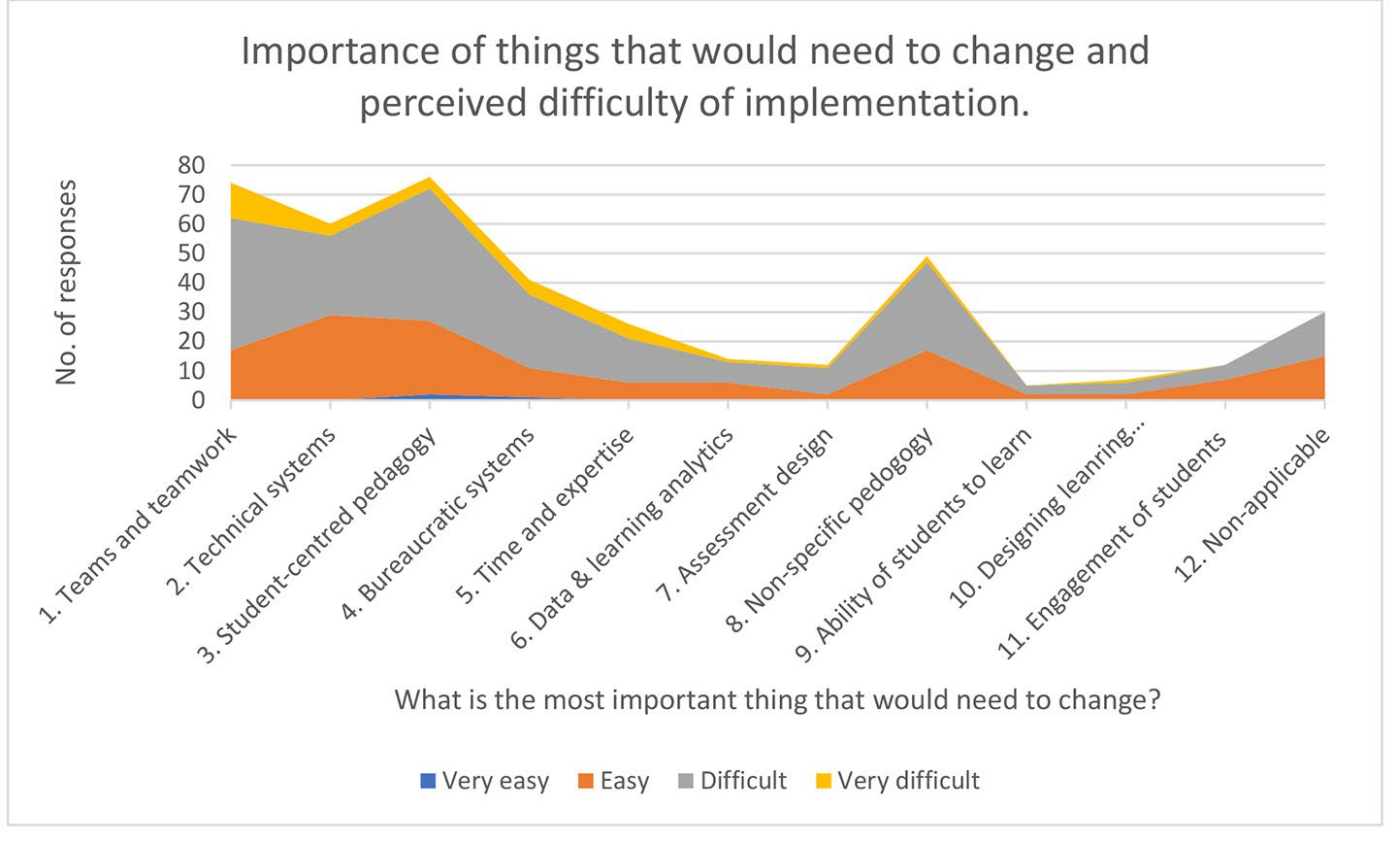
Combination of ‘what is the most important thing that would need to change’ by ease/difficulty of implementation

The results of the two questions posed by Instrument A are combined and represented in Fig. 1. It suggests that ‘student-centred pedagogy’ and ‘establishing and operating teams for design purposes’ were considered the two the most important things that would need to change for the participants working in this context. Substantial amounts of responses also referenced other important things that would need to change. Almost a third of responses referenced some form of teaching and learning pedagogical change (combination of 3, 7, 8 and 10) as being important.

The findings also suggest that the participants considered ‘establishing and operating teams’ and ‘bureaucratic systems’ to be the most difficult of the things that would need changing. Around 3 times as many responses in these categories also responded with ‘difficult/very difficult’ as compared with ‘very easy/easy’ (giving a ratio of 3:1). The ratio for changing to ‘student-centred pedagogy’ or any kind of pedagogical change was around 2:1, whilst the ratio for changes to ‘technical systems’ was 1:1, which indicates these were perceived to be easier to implement.

### Strategies for Encouraging the Adoption of Constructivism and Student-focused Learning in the LDCC Workshop

The co-designers of the LDCC Workshop responded to this feedback by adopting strategies designed to address the challenges identified above.   .

For example, to address the establishment and operation of teams participants are allocated into teams of five by the host institution and these teams work collaboratively throughout the whole PD programme of activities. By doing this participants gain experience of a constructivist strategy that facilitates establishing meaning through social negotiation (Ertmer & Newby, 2013) . Allocation is made to maximise differences in subject expertise, role and experience and encourage participants to be exposed to people from within their institution who they would not normally work with. Teams are presented with the practical, real-world challenge of designing and creating an ODL course together. Structured activities are introduced to guide the teams in making grounded decisions about the ODL course they will design. For example, teams engage in debate about the course subject area, duration and level. They then allocate design roles and responsibilities to one another in line with self-declared skills, interests and competencies. Throughout the LDCC Workshop, teams spend at least 20% of their time discussing and critiquing the LDCC approaches presented to them, establishing their own views and constructing contextualised knowledge and meaning.

The participants practice student-focused learning by using a bespoke online tool to decide on three words that would like a student to use to describe the course they are designing once it is completed (LD Vision Statement Tool, 2021). The participants build a vision statement in the form of a social media post from the key words they decided on which represents the student experience from the perspective of the student and will go on to guide the design (Olney et al, 2019). Teams also consider the needs, characteristics and learning preferences of their hypothetical students by creating one or more student profiles. A bespoke structured activity guides and prompts reflection in the teams about who their students will be, and how they can design for them which has also been seen to be effective in an African context (Mittelmeier et al., 2018).

As a design fundamental, constructive alignment also forms an important pillar of the LDCC Workshop both as a conceptual way to embed constructivism in design (Biggs & Tang, 1996) and also in the practical ‘construction’ of curriculum (Loughlin et al., 2021). In line with Moon (2002), the LDCC Workshop situates constructive alignment as a framework within which to design and balance student-focused intended learning outcomes (ILO), activities and assessment tasks that enable the student to demonstrate these ILO, rather than being able to recite ‘correct’ memorised information or knowledge for high-stakes exams such as the *gaokao* (national college entrance exam), which are common in Chinese traditional education (Tan, 2017).

The learning designs created by the LDCC participants is iteratively structured and visualised on an Activity Planner using the Activity Types Classification Framework (Conole, 2013) and the expected student workload allocated (Olney et al., 2019). This framework requires teams to categorise the proposed activities depending on what the student is actually doing at that time, rather than at a cognitive level, and therefore places the student experience front and centre in the designers’ thoughts (Olney et al.., 2019). Research from the UKOU suggests that the introduction of this approach to design for ODL practice has led to educators designing less assimilative, traditional teaching content and including more ‘active learning’, around building skills in communication, interactivity and experiential learning common in student focused approaches (Toetenel & Rienties, 2016).

To further enhance the constructivist concepts of relevance, practicality, and contextualised application (Ertmer & Newby, 2013) participants are given access to their own Virtual Learning Environment (VLE) to transfer the learning design they have agreed on in the Activity Planner onto the online VLE website and experience the challenges this can bring. ODL courses designed in the LDCC Workshop commonly have an initial layout of three to five weeks’ worth of study and this output forms the basis of the final presentation to peers.

### Instrument B: Measuring Impact

#### Changes to the Constructivist and Student-focused Learning Practice of the Participants

Table 2 shows that 92 (69%) of the participants who responded to Instrument B said they had implemented at least one LDCC approach at some point since the LDCC Workshop. Of these 92, 61 also referenced LDCC approach(es) they had implemented. Table 3 summarises these 119 separate, coded references. It shows that the most heavily referenced LDCC approach that was implemented was ‘student-centred pedagogy’. Typical comments included, ‘the student-centred concept is being emphasised in all elements of course design’ [041], ‘course design is now student- centred’ [126] and ‘design learner-centred teaching activities’ [068]. In contrast, there were relatively few references to the implementation of ‘establishing and operating teams for design purposes’, ‘technical systems’ or ‘bureaucratic systems’ despite these previously having been previously identified as important components of change (Fig. 1).

Table 4 suggests that whilst the respondents recognise a wide-range of benefits of implementing the LDCC approaches into their practice, by far the largest perceived benefit (119 from 134 responses, or 89%) was ‘designing student-centred learning’.

#### Strategies for Supporting Changes to the Task Perception and Subjective Educational Theory of the Participants in the LDCC Workshop

The LDCC Workshop uses a series of presentations that outline LDCC approaches that make up the role of design for ODL educators at the UKOU. Collectively, these can be viewed as concrete set of norms which provide an answer to the questions; ‘what must I do to be a proper ODL teacher’ and ‘how can I become a proper ODL teacher?’ – questions that influence the *task perception* and *subjective educational theory* of teachers. This is not to say that LDCC approaches are the most valid, or best practice, or should be adopted, only that they exist in the context of the UKOU and have been summarised and presented in an appropriate way for consideration by others.

To surface and share views participants have repeated opportunities to critique, debate and elaborate in their teams on the value they place on LDCC approaches in their own context whilst designing and creating the ODL course. The participants make their design decisions and judgements explicit and he efficacy of each is tested through discussion. Acceptance of LDCC approaches in their entirety is actively challenged by the facilitators and opportunities for adaption is encouraged. Further, to assist in refining and extending the *subjective educational theory* of the participants, the LDCC Workshop utilises key reflection points as activities which provide ‘an important agenda’ for PD and can be used to help manage the challenges that educational change, such as implementing ODL, brings (Kelchtermans, 2009: 264).

For example, the completed Activity Planner which classify activities by Activity Type and expected student workload, generate a data set that is representation of the learning design. This design is used as a prompt to reflect on the student experience that has been created and the strategies adopted to achieve it (Dalziel et al., 2016; Olney et al., 2019). Learning Analytics evaluation and feedback plans, developed via scenario- based activities, and the final presentation are used as other reflective prompts.

It is acknowledged that changes to the *task perception* and *subjective educational theory* of the participants, and the impact of the LDCC Workshop on them, is not easy to measure in a simple self-reporting survey. However, 129 from 134 participants (96%) responded that the LDCC Workshop had changed their way of thinking about designing ODL either ‘moderately’ (38), ‘quite a lot’ (51) or ‘a lot’ (40) (Table 5).

The percentage of those who had implemented any LDCC approach was progressively higher amongst those who perceived they had changed their design thinking to a greater extent. In Table 5, 38 participants said they had a ‘moderate’ change in design thinking and, of these, 19 also referenced implementing at least one LDCC approach (50%). 51 participants said they had ‘quite a lot’ of change in design thinking’ and, of these, 34 also referenced implementing at least one LDCC approach (67%). 40 participants said they had ‘a lot’ of change in design thinking and, of these, 37 also referenced implementing at least one LDCC approach (93%). These findings suggest a positive relationship between extent of implementation and changes to thinking about how to design ODL.

There is also tentative evidence in the impact of the LDCC Workshop on the fundamental beliefs of teachers to be found in the qualitative comments. For one participant LDCC approaches represented ‘a big change in ideas and concepts’ [95]. Other qualitative impact related comments referenced ‘broadening’ learning horizons [06] [27] [10], improving a personal ‘teaching philosophy’ [50] and helping to ‘clear my mind on many questions that had troubled me before, for example, the purpose of course design’ [29].

**Fig. 2:**
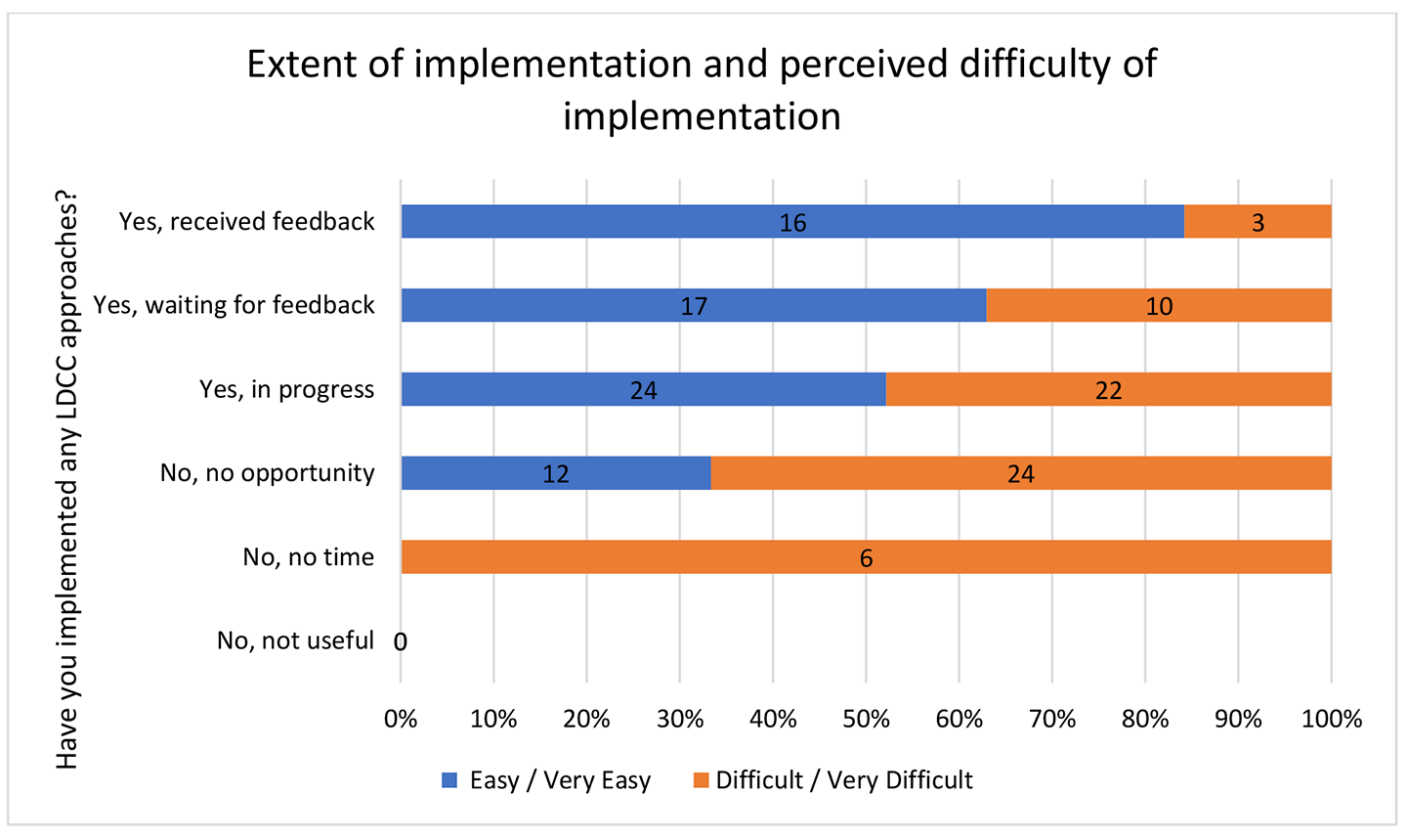
The extent of implementation and perceived difficulty of implementation

In designing an ODL course during the PD the experiential nature of the LDCC Workshop is designed to facilitate discussion within teams about when, how and in what circumstances LDCC approaches might ‘work for them’ or be ‘true to their practice’ and take root. Figure 2 combines the results of two questions from Instrument B about extent of implementation and perceptions of ease/difficulty of implementation. It suggests that for the participants the perceived difficulty of implementation reduced the more experience they had of implementing the LDCC approaches in their practice.

In choosing to go on and implement some LDCC approaches or not, the participants exercised value judgements about ‘what works for them’ and what maybe ‘true for their practice’. Taken together the findings show that some participants, for example, considered student-focused learning to be important, practised this during the PD, went on to implement this into their own practice, and consequently changed their perception of difficulty of implementation. Further, it seems reasonable to suggest that this experiential pedagogy was able to shift some participants towards a different understanding of their own *task perception* and/or *subjective educational theory.* However, this assertion would require a more detailed qualitative conversation to confirm this was the case.

Establishing and operating teams and teamwork is a fundamental element in raising the quality of course design in education (Schmidt et al., 2016; Chao et al., 2010; Galley, 2015; Halupa, 2019). The evidence collected from Instrument A immediately after the LDCC Workshop took place, suggests that in general the participants perceived implementing the constructivist approach of ‘teamwork’ as being an important thing to change but also that it represented a more difficult challenge than implementing ‘student-centred learning’ (Fig. 1). After some time had passed, participants then reported in reasonable numbers that they perceived LDCC approaches could effectively support them to better collaborate with colleagues (Table 3), but very few participants reported implementing ‘teamwork’ into their practice (Table 2).

It seems possible that an explanation for this could be found in the *task perception* of the participants themselves. As has already been established, LDCC approaches provide a UKOU subjective interpretation of what being ‘a proper ODL teacher’ is, but it is likely this is substantially different to the reality that the Chinese ODL teachers encounter in their daily practice. They may have little or no agency over the way their job is organised or clarity about their role and responsibility, especially regarding teamwork. One participant noted, ‘The training with the UKOU is very rewarding and inspiring, but it is difficult to put what I have learned into practice in teaching and course design after returning to China’ [54]. The relatively higher levels of implementation of student-focused learning could suggest the participants have more agency over what is taught and how it is taught than how teams are established in their institution (Prosser & Trigwell, 2011). This warrants further investigation.

### Limitations and Further Study

The respondents to the two instruments were self-selecting, and the study did not control for any variables such as gender, institution, age or seniority. Names were not collected and so it was not possible to connect responses from instrument A directly with instrument B. Both instruments were straightforward and uncomplicated to maximise levels of engagement. Whilst usable data was substantial (254 responses for Instrument A, 134 for Instrument B) the actual response rate for instrument B was 25% and demonstrates the difficulty in collecting data after an event. The time between attending the PD and completing instrument B was not consistent and may have influenced the results.

The findings suggest that further study is both viable and desirable, and the authors are currently interviewing participants to explore further their reflections on this PD, their implementation of LDCC approaches and the interaction between context, extent of implementation and changes to *task perception* and *subjective educational theory.* Findings from this study were vital in developing the interview instrument and shaping the nature of that study. The hope is that future study will uncover more detailed observations and further inform PD for the design of ODL, either in UK based or international settings.

## Conclusion & Recommendations

Drawing on recent literature and the results from two instruments, this study set out to explore the research question:

*Can PD, which aligns LD frameworks with constructivist and student-focused pedagogies, support the changing professional identities of teachers when they are tasked with designing ODL?*

Analysis of the findings using the selected methodology suggests that that the described approach to PD has been successful in supporting the changing professional identities of teachers when tasked with designing ODL. This has two major implications for PD providers tasked with responding to the global demand for PD for the design of ODL.

Firstly, from a practice perspective, the findings show that a substantial amount of participants went on to implement at least one LDCC approach in their practice. This success is important since recent literature has suggested that substantial gaps exist between the perceived skills and competencies of educators to design and implement ODL approaches, and the PD available to them. A significant proportion of participants also believed the LDCC Workshop prepared them to implement ‘student-focused learning’ most effectively, and that they went on to implement this approach to a greater extent than other approaches they also previously considered to be important. The contention posed here is that the participants were able to do this because they were provided with a constructivist environment in which to practise, debate and reflect on the value of a set of LDCC approaches (or norms) currently in use at the UKOU which repeatedly modelled the concept of ‘student-focused learning’. In response to calls from previous research, these normative practices were described in detail and provide a valuable reference. Therefore, it is recommended that other PD providers could build on this finding and, using the model outlined here, combine a set of normative design for ODL practices from their own institution, with a team-based, collaborative environment, in which participants can construct knowledge together and develop their own contextualised design for ODL practice.

Secondly, from a methodology perspective, both *task perception* and *subjective educational theory* provided a useful framework which allowed for an articulation of the relationship between the pedagogy of the LDCC Workshop and the impact on the professional teaching identity of the participants. To the best of our knowledge this is the first time that these well-established concepts have been applied to PD for the design of ODL. Whilst progress was indicated by some participants in conceptualising what it means to be a proper ODL teacher in their own context, and how to realise that aspiration, the instruments used for the collection of data adopted here could be expanded. Other researchers could consider a more qualitative approach using semi-structured interviews to measure the impact of their PD for the design of ODL on the professional teaching identities of the participants who attend. Since recent literature reviews suggest that supporting changes to the professional teaching identities of teachers is paramount in PD for the design of ODL, it is recommended that other providers utilise *task perception* and *subjective educational theory* when developing and evaluating PD programs.

## Data Availability

The datasets used and or analysed during this study are available from the corresponding author on reasonable request.
